# P-1101. Can targeted interventions improve the hand hygiene compliance among Healthcare Workers in low resource settings?

**DOI:** 10.1093/ofid/ofaf695.1296

**Published:** 2026-01-11

**Authors:** Md Shariful Amin Sumon, Fairoze Masuda Akther, Tanjeemay Tamanna, Antara Swarnali Priyanka, Syed Abul Hassan Md. Abdullah, Aninda Rahman, Md Saiful Islam, Md Golam Dostogir Harun

**Affiliations:** icddr,b, Dhaka, Dhaka, Bangladesh; icddrb, Dhaka, Dhaka, Bangladesh; icddr,b, Dhaka, Dhaka, Bangladesh; icddr,b, Dhaka, Dhaka, Bangladesh; SafetyNet, Dhaka, Bangladesh, Dhaka, Dhaka, Bangladesh; Directorate General of Health Services, Bangladesh, Dhaka, Dhaka, Bangladesh; UNSW, Sydney, New South Wales, Australia; icddrb, Dhaka, Dhaka, Bangladesh

## Abstract

**Background:**

Hand hygiene (HH) is a crucial component of infection prevention, and adherence to proper HH practices is often below optimal levels in many healthcare settings in low- and middle-income countries (LMICs). Interventions focused on behavior change and increasing awareness could enhance hygiene practices among healthcare workers (HCWs). This study measures hand hygiene practices among HCWs in targeted intervention wards compared to non-targeted intervention wards in tertiary hospitals in Bangladesh.

**Figure:**
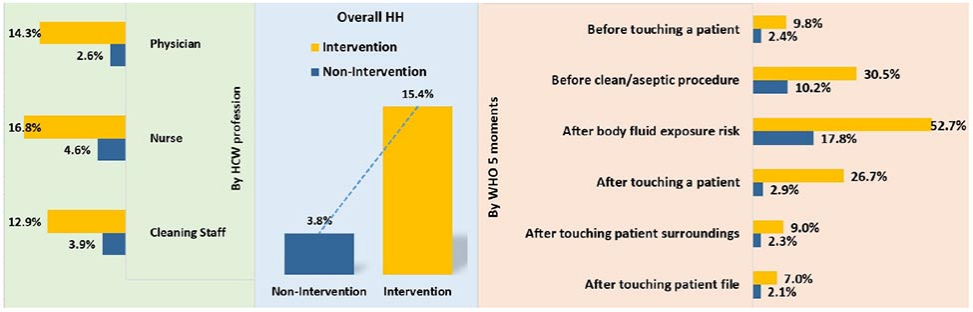
HH compliance among healthcare workers following interventions

**Methods:**

From September to December 2024, we conducted an observational study in six tertiary care hospitals. Each hospital had eight wards: four intervention and four non-intervention wards. HH compliance among physicians, nurses, and cleaning staff was assessed through direct observation based on the WHO's five Moments for HH guidelines. The multifaceted intervention included training and education, visual reminders, and real-time feedback mechanisms. HH compliance was defined as the correct HH actions over the observed opportunities.

**Results:**

A total of 10,132 HH opportunities were observed. Overall, HH compliance was significantly higher in intervention wards (15.4%, 522/3,388), compared to non-intervention wards (3.8%, 256/6,744). Among HCWs in intervention wards, nurses had the highest compliance at (16.8%, 292/1,735), followed by physicians at 14.3%, 176/1,234) and cleaners (12.9%, 54/419). In non-intervention wards, compliance among all HCWs was alarmingly low. The highest compliance in intervention wards was observed after exposure to risk from body fluids (52.7%), followed by before aseptic procedures (30.5%), and after touching a patient (26.7%). In contrast, compliance for before patient contact (9.8%), after touching patient surroundings (9.0%), and after touching patient files (7.0%) was lower but higher than non-intervention wards (2.4%, 2.3%, and 2.1%, respectively).

**Conclusion:**

The intervention led to significant improvements in HH practices among all HCWs groups at each WHO moment, compared to units without the intervention. However, the overall adherence remained low. These findings underscore the importance of policy enforcement including structured education and behavior-focused interventions to enhance compliance.

**Disclosures:**

All Authors: No reported disclosures

